# Contrasting Roles of Deoxynivalenol and Nivalenol in Host-Mediated Interactions between *Fusarium graminearum* and *Sitobion avenae*

**DOI:** 10.3390/toxins8120353

**Published:** 2016-11-30

**Authors:** Jassy Drakulic, Mohd Haziq Kahar, Olubukola Ajigboye, Toby Bruce, Rumiana V. Ray

**Affiliations:** 1 Division of Plant and Crop Sciences, University of Nottingham, Sutton Bonington Campus, College Rd., Sutton Bonington LE12 5RD, UK; jassydrak@gmail.com (J.D.); mhaziq79@gmail.com (M.H.K.); bukky.ajigboye@nottingham.ac.uk (O.A.); 2 Biological Chemistry and Crop Protection, Rothamsted Research, Harpenden Herts AL5 2JQ, UK; toby.bruce@rothamsted.ac.uk

**Keywords:** deoxynivalenol, nivalenol, trichothecenes, Fusarium head blight, aphids, volatiles

## Abstract

*Fusarium graminearum* is the predominant causal species of Fusarium head blight in Europe and North America. Different chemotypes of the species exist, each producing a plethora of mycotoxins. Isolates of differing chemotypes produce nivalenol (NIV) and deoxynivalenol (DON), which differ in toxicity to mammals and plants. However, the effect of each mycotoxin on volatile emissions of plant hosts is not known. Host volatiles are interpreted by insect herbivores such as *Sitobion avenae*, the English grain aphid, during host selection. Previous work has shown that grain aphids are repelled by wheat infected with DON-producing *F. graminearum*, and this study seeks to determine the influence of pathogen mycotoxins to host volatile chemistry. Volatile collections from infected hosts and olfactometer bioassays with alate aphids were performed. Infections with isolates that produced DON and NIV were compared, as well as a trichothecene deficient transformant derived from the NIV-producing isolate. This work confirmed the repellent nature of infected hosts with DON accumulation. NIV accumulation produced volatiles that were attractive to aphids. Attraction did not occur when NIV was absent and was, therefore, a direct consequence of NIV production.

## 1. Introduction

Fusarium head blight (FHB) is a fungal disease caused by a complex of pathogens from the genus *Fusarium*, and *F. graminearum* is the most prevalent species in North America [1] and Europe [2,3] at present. As a result of FHB disease, cereal host crops become contaminated with mycotoxins, grain quality is impaired, and yield is lost [4]. Trichothecene mycotoxins produced by *Fusarium* species are a group of toxic chemicals formed from tetracyclic sesquiterpenoids with an epoxy-ring at the C12,13 position and can be classed into Type A or B depending on the absence or presence, respectively, of a ketone functional group at the C8 position and a hydroxyl group at C7. *F. graminearum* produces Type B trichothecene mycotoxins, including deoxynivalenol (DON) and nivalenol (NIV) and acetylated derivatives of these: 3-ADON, 15-ADON, and 4-ANIV [5]. A legal limit for DON has been set in Europe for different categories of grain products, with a maximum of 1250 μg·kg^−1^ DON in wheat grain intended for human consumption [6]. 

The prevalence of different *F. graminearum* chemotypes varies according to geographical region, with NIV chemotypes prevailing in Asia and DON chemotypes prevailing in Europe and North America [7,8]. The geographical distribution of the different chemotypes has also been recently described to be shifting, although the factors driving the change are not known. Among the DON producing strains, these can be categorised into those producing 3-ADON and 15-ADON. In North America, 15-ADON isolates are traditional, although emerging populations of the 3-ADON type or of the 15-ADON type with genetic similarities to the 3-ADON type have been observed [9,10]. Shifting patterns of mycotoxin production by this species show that ongoing study into the impact of the different chemotypes on hosts and their environment is needed. 

The mycotoxins produced differ in phytotoxicity [11] and toxicity to mammalian consumers [12]. The mode of action for all is to inhibit protein synthesis by interacting with small 60S ribosomal subunits and peptidyltransferase enzymes. In mammals, the health implications of consuming DON and NIV include feed refusal and weight loss, plus DON has been reported to have immunosuppressive effects and NIV to cause oral lesions [13]. In plant hosts, the phytotoxicity has been measured by using different model systems. Wheat coleoptile growth was severely inhibited by DON, whereas the effect of NIV was no different to negative controls [11]. In *Chlamydomonas reinhardtii*, toxicity of NIV was detectable, but less still than that of DON [14]. Neither of these studies truly demonstrates the phytotoxic effects to cereal spikes at the site of head blight disease, but indicates that the phytotoxicity of NIV is less than that of DON and is host dependent. 

The trichothecene biosynthetic pathway is governed by *Tri* genes, the first in the pathway being *Tri5* which encodes trichodiene synthase which catalyses the conversion of farnesyl pyrophosphate into trichodiene [15]. The genetic difference of *F. graminearum* chemotypes has been elucidated [5]; NIV producers differ from DON producers in their presence of functional *Tri13* and *Tri7* genes, which carry out hydroxylation and acetylation of the C4 position of the trichothecene ring [5,16]. In the absence of these functioning genes, the trichothecene biosynthetic pathway is diverted at this point in DON-producing strains. To determine the role of mycotoxins as virulence factors, DON- and NIV-producing isolates were previously transformed to knock out the *Tri5* gene, and these mutants were shown to be unable to produce any mycotoxins [17,18]. Growth of *Tri5* mutants was impeded at the rachis node, indicating that DON assists spread of the fungus into other spikelets of the spike [19]. DON has been shown to be an important virulence factor in the colonisation of wheat by *F. graminearum*, whereas NIV is a more important virulence factor in the infection of maize by this species [18]. Accordingly, different chemotypes can have different pathogenicity depending on the host infected, although NIV-producers in some cases have been shown to be less pathogenic to wheat than DON-producers [7]. DON has been shown to be able to travel in phloem sieve tubes and xylem vessels in wheat spikes infected with *F. culmorum* [20], occurring in tissues beyond those colonised by fungal hyphae. DON has specifically been linked to bleaching of spikes upwards from the site of infection [21], which is less commonly observed with NIV-producing *F. graminearum* infection [22]. 

Interactions can occur between arthropods and host plants infected with FHB [23], but information is lacking on the impact of the mycotoxin output of the infecting fungi on visiting herbivores. A previous study of wheat spikes infected with *F. graminearum* showed that the volatile chemical emissions were repellent to English grain aphids, *Sitobion avenae* Fab. (Hemiptera: Aphididae), in olfactometer bioassays [24], and it was hypothesised that this effect is driven by the mycotoxin output of the pathogen. Thus, a series of volatile collection experiments were performed to test this hypothesis. Isolates of *F. graminearum* of known chemotype described previously [18], along with *tri5* knock-out transformants of the NIV-producing isolate, were used to infect wheat spikes and volatile chemicals were collected at five time points from 6 h to 7 days after inoculation. The aims of this work were to compare the volatile chemical emissions of NIV and DON producers, and a NIV trichothecene deficient mutant, then to examine the behavioural response of aphids, *Sitobion avenae*, to those volatiles. Visual disease assessment, real-time PCR, and mycotoxin extraction were used to validate the treatments. 

## 2. Results

### 2.1. Visual Disease Assessment

Wheat spikes were treated in one of four ways: point inoculated with DON-producing (DON-wt), NIV-producing (NIV-wt), or NIV-deficient knock-out mutant (NIV-ko) *Fusarium graminearum* isolates, or with sterile water (NTC). Disease severity was assessed as the proportion of total spikes displaying any symptoms (either dark, water-soaked lesions or bleached spikelets), and bleaching severity was recorded as the proportion of spikelets that became bleached (either following the presence of a lesion or due to ‘wilt’ above the point of infection).

**Figure 1:**
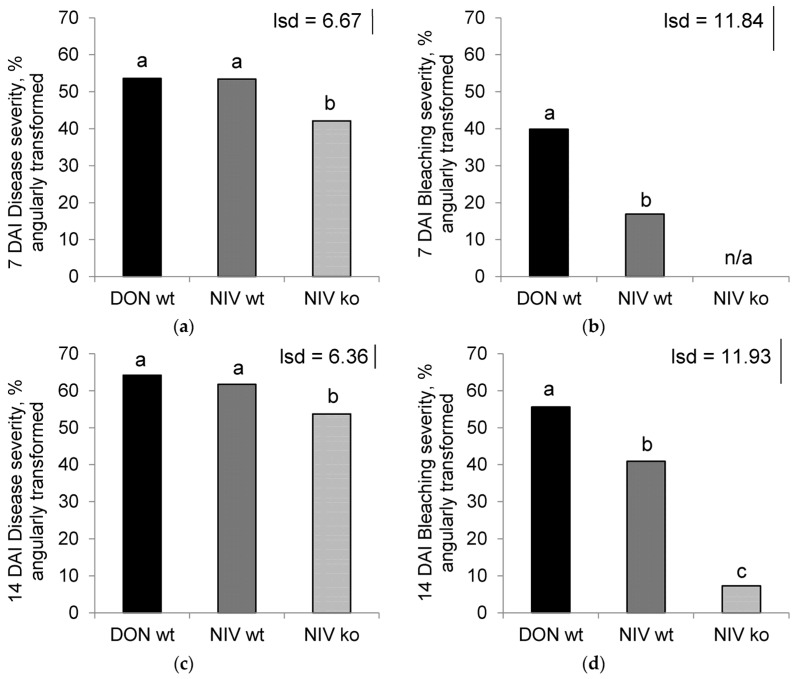
Severity at 7 days after inoculation (DAI) of (**a**) disease (*p* < 0.001) and (**b**) bleaching (*p* < 0.001); and severity at 14 DAI of (**c**) disease (*p* = 0.005) and (**d**) bleaching (*p* < 0.001). Disease severity shows the percentage of spikelets with lesions and/or bleaching; bleaching severity shows the percentage of spikelets with bleaching. All data were angularly transformed. DON-wt: deoxynivalenol (DON)-producer; NIV-wt: nivalenol (NIV)-producer; NIV-ko: *tri5* knock out transformant of NIV-wt. Data marked with different letters are significantly different (lsd; *p* = 0.05). NIV-ko data for bleaching severity at 7 DAI was zero and, therefore, excluded from analysis.

Treatment was a significant factor accounting for disease severity at 7 days after inoculation (DAI) (*p* < 0.001), 14 DAI (*p* = 0.005) and bleaching severity at 7 DAI (*p* < 0.001) and 14 DAI (*p* < 0.001) (Figure 1). Generally, disease and bleaching severity were higher in the toxin-producing DON-wt and NIV-wt treatments and lower in the NIV-ko treatment, whilst controls showed no symptoms or bleaching whatsoever. At 7 DAI (Figure 1A), the disease severities were similar for DON-wt (53.6%) and NIV-wt (53.4%) but significantly fewer symptoms were observed in the NIV-ko treatment (42.1%) (Figure 1A). Bleaching at 7 DAI (Figure 1B) was significantly greater in DON-wt (39.9%) than NIV-wt (16.9%) and no signs of bleaching were observed in NIV-ko plants. At 14 DAI the disease severity (Figure 1C) had increased, with similar levels in DON-wt (64.2%) and NIV-wt (61.7%) with significantly less disease observed in NIV-ko (53.7%). Bleaching at 14 DAI (Figure 1D) also showed significantly greater severity in the DON-wt treatment (55.6%) than what was observed in NIV-wt (40.9%). At this time point, NIV-ko showed some bleaching (7.3%) which was significantly less than the NIV-wt.

### 2.2. Characterisation of Isolates by QPCR

**Figure 2:**
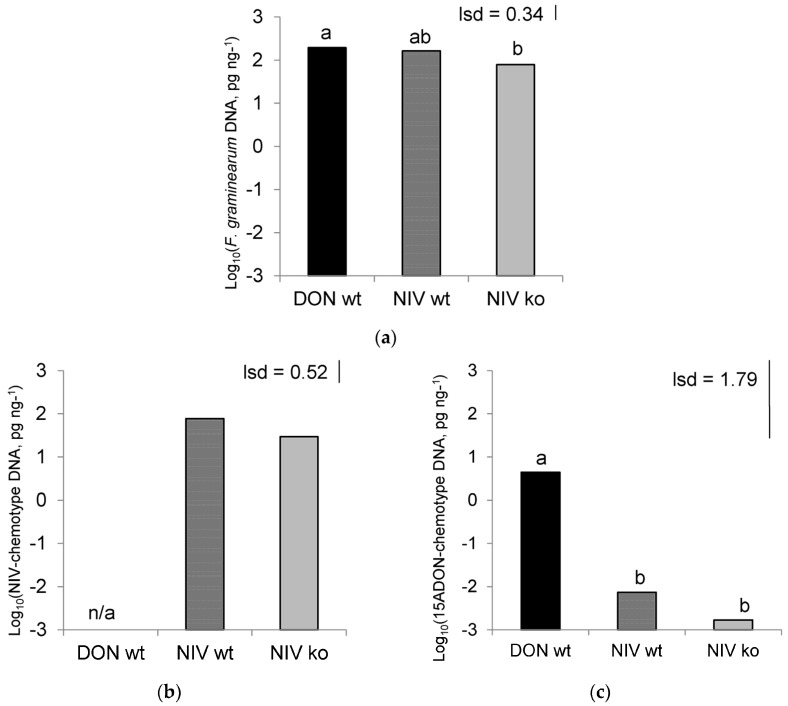
Fungal biomass quantified using Real-time QPCR with primers specific for (**a**) *F. graminearum* (*p* = 0.06); (**b**) NIV-chemotype isolates, based on *Tri12* (*p* = 0.096); and (**c**) 15ADON-chemotype isolates, based on *Tri12* (*p* = 0.003). Data log_10_ transformed. DON-wt: DON-producer; NIV-wt: NIV-producer; NIV-ko: *tri5* knock out transformant of NIV-wt. Data marked with different letters are significantly different (least significant difference (lsd); *p* = 0.05). DON-wt data were zero for NIV DNA so were excluded from analysis.

Four Real-time quantitative polymerase chain reaction (QPCR) assays were performed to quantify the concentration of five different sections of DNA in the flour from treated ears, which are detailed in Table 1. Primers with different specificities were used to characterise the concentration of their targets in DNA extracts from treated wheat spikes, as an indication of fungal biomass for the different strains used. When using *F. graminearum* specific primers, target sequences were amplified in all infected samples and below the limit of detection in controls. Treatment was a significant factor at the 10% level (*p* = 0.06) in accounting for the differing concentrations of target DNA. When treatment means were compared with the 5% least significant difference (lsd) value, the concentration in spikes treated with DON-wt were significantly higher than in those treated with the NIV-ko strain (Figure 2A), and NIV-wt treated samples contained an intermediate concentration of *F. graminearum* DNA. 

An assay using primers specific for sequences within the *Tri12* gene that are characteristic of NIV-producing isolates amplified its target in NIV-wt and NIV-ko samples, but target DNA was below the limit of detection in the remaining two treatments. The difference in concentration of NIV-producer DNA between NIV-wt and NIV-ko treatments only differed at the 10% level (*p* = 0.096) with the greater concentration occurring in the NIV-wt treatment (Figure 2C). 

The third assay used primers that target *Tri12* sequences characteristic of 15ADON-producers. The concentration of target DNA amplified in this assay differed significantly between treatments (*p* = 0.003). DON-wt samples accumulated significantly more target DNA compared to that of NIV-wt and NIV-ko (Figure 2D). The presence of DON-chemotype DNA in NIV-chemotype isolate infection was unexpected and presumed to be due to a low level of contamination of host plants. A final assay using primers specific for DNA sequences found in 3ADON-producers showed no significant difference in the concentration of target DNA between treatments, with a (back-transformed) grand mean of 3ADON-producer DNA across all treatments of 0.001 pg·ng^−1^.

### 2.3. Characterisation of Toxin Profiles of Isolates by LCMS/MS

**Figure 3:**
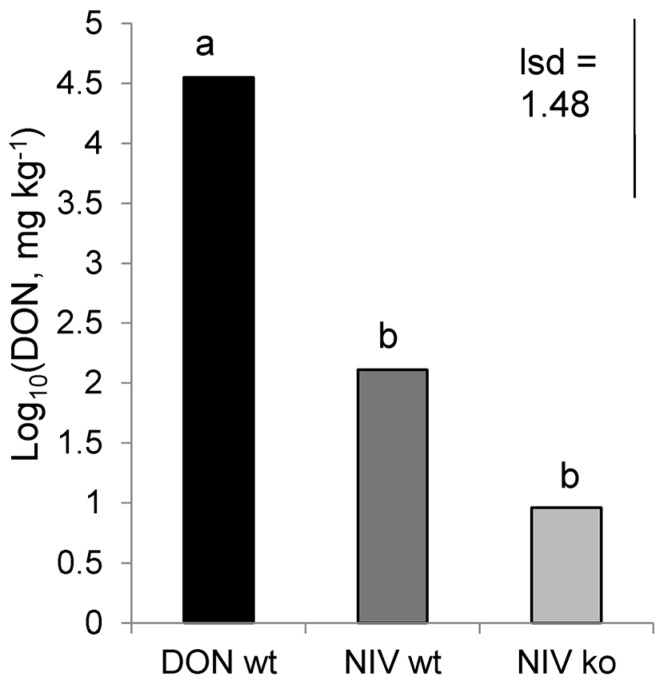
Concentration of DON (log_10_ transformed) in ears treated with *F. graminearum* of different chemotypes (*p* < 0.001). DON-wt: DON-producer; NIV-wt: NIV-producer; NIV-ko: *tri5* knock out transformant of NIV-wt. Data marked with different letters are significantly different (lsd; *p* = 0.05).

**Figure 4:**
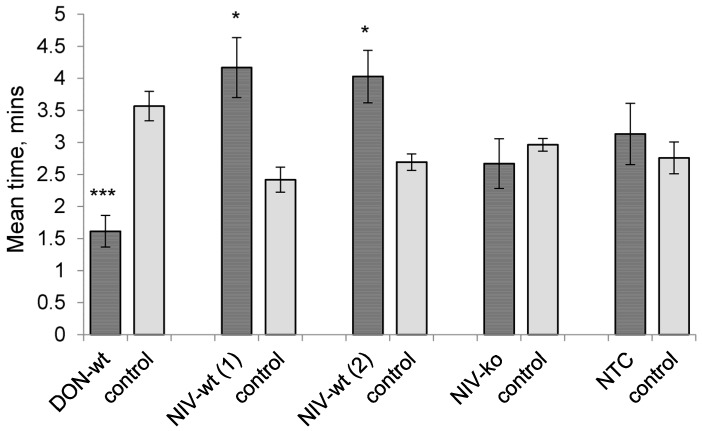
Behavioural responses of *Sitobion avenae* to volatile chemicals sampled from *Fusarium*-infected hosts. DON-wt: DON-producer; NIV-wt: NIV-producer; NIV-ko: *tri5* knock out transformant of NIV-wt; NTC: non-treated control. *** *p* < 0.001, * 0.01 ≤ *p* < 0.05.

DON concentration was significantly affected by treatment (*p* < 0.001) with the greatest concentration in DON-wt which had a back-transformed mean of over 35,000 mg·kg^−1^. While there was low level contamination by the DON-producing isolate in plants infected with NIV-wt and NIV-ko, these treatments contained significantly less DON than DON-wt (Figure 3) and contained just 128 and 9 mg·kg^−1^ (back-transformed means), respectively, for each of NIV-wt and NIV-ko. The amounts of DON in mock-inoculated controls were lower than the limit of detection. The concentration of NIV was below the limit of detection in all treatments except for NIV-wt samples, which accumulated a mean concentration of 115.2 mg·kg^−1^. While there were different extraction methods for DON and NIV, limiting the comparability of the concentration values for each toxin to the other, it is worth noting that for the two replicates whose volatile emissions were used in aphid olfactometer assays, the ratio of NIV:DON contamination was approximately 300:1 for the replicate referred to as NIV-wt (1) (Figure 4) and 35:1 for NIV-wt (2). 

### 2.4. Behavioural Responses of Sitobion avenae to Host Volatiles

Alate aphids spent significantly less time in areas of the olfactometer treated with volatiles sampled from hosts inoculated with the DON-wt isolate compared to control areas (*p* < 0.001), showing a repellent response. In contrast, aphids were attracted to volatiles from hosts inoculated with the NIV-wt isolate as they spent significantly more time in areas treated with infected host volatiles compared to control areas in two repeats of the experiment (*p* = 0.011; *p* = 0.010). Volatiles from both mock-inoculated hosts and hosts inoculated with the NIV-ko isolate had no significant influence on aphid behaviour and there was no difference in mean time spent in treated areas compared to solvent-only controls for either treatment (Figure 4).

### 2.5. Chemical Composition of Host Volatiles

**Figure 5:**
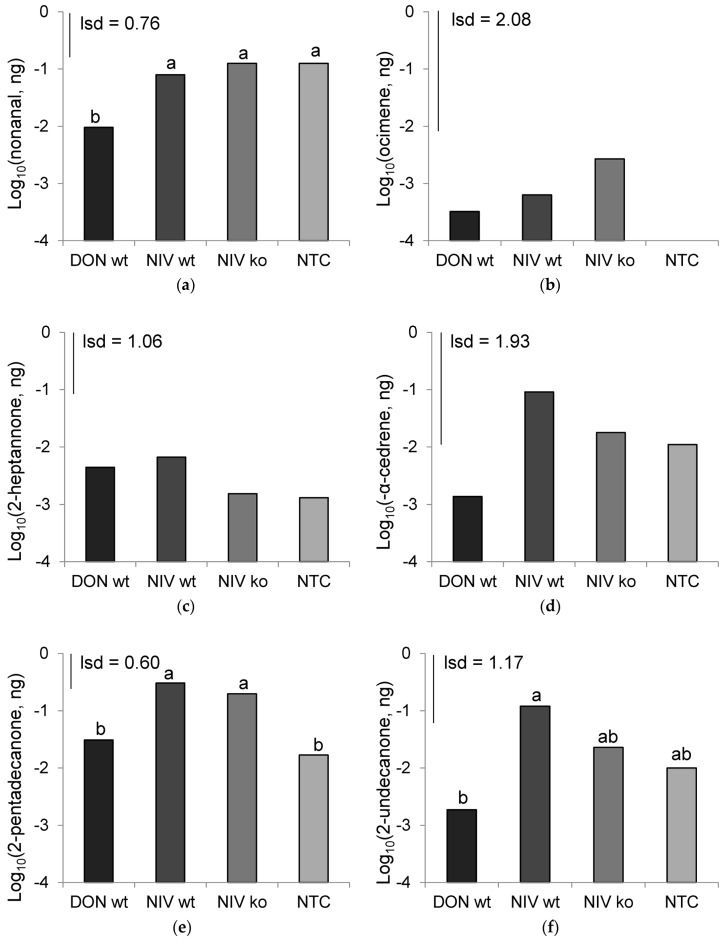
Mean abundances of volatile chemicals, log_10_ transformed, across all time points. (**a**) Nonanal (*p* = 0.004); (**b**) ocimene (ns); (**c**) 2-heptanone (ns); (**d**) α-cedrene (*p* = 0.015); (**e**) 2-pentadecanone (*p* < 0.001); (**f**) 2-undecanone (*p* < 0.001). DON-wt: DON-producer; NIV-wt: NIV-producer; NIV-ko: *tri5* knock out transformant of NIV-wt; NTC: non-treated control. Data marked with different letters are significantly different (lsd; *p* = 0.05).

A shortlist of commonly occurring chemicals were provisionally identified from chemical libraries and co-injected with a host volatile sample to confirm the identification by peak enhancement gas chromatography mass spectrometry (GC/MS). By this process, 2-pentadecanone, 2-heptanone, 2-undecanone, nonanal, and (-)α-cedrene were confirmed, and abundance data for this shortlist was collected. The mean chemical abundances collected at 7 days after inoclulation differed significantly between treatments for 2-pentadecanone (*p* = 0.003), 2-undecanone (*p* = 0.04), and nonanal (*p* = 0.025), but (-)α-cedrene, ocimene and 2-heptanone did not (Figure 5). 2-pentadecanone was most abundant in the NIV-wt treatment (0.305 ng·μL^−1^) which was similar to that of NIV-ko (0.198 ng·μL^−1^), and both were significantly greater than the concentration in the DON-wt treatment (0.030 ng·μL^−1^) and mock-inoculated controls (0.017 ng·μL^−1^). 2-undecanone was also greatest in the NIV-wt treatment (0.120 ng·μL^−1^), which contained a significantly greater abundance than the DON-wt (0.002 ng·μL^−1^). NIV-ko (0.023 ng·μL^−1^) and mock-inoculated controls (0.010 ng·μL^−1^) contained an intermediate concentration of 2-undecanone. Nonanal abundances were the greatest in mock-inoculated samples (0.126 ng·μL^−1^) and NIV-ko samples (0.126 ng·μL^−1^), which were statistically similar to the concentration in NIV-wt (0.079 ng·μL^−1^), and all were significantly greater than DON-wt (0.010 ng·μL^−1^).

**Figure 6:**
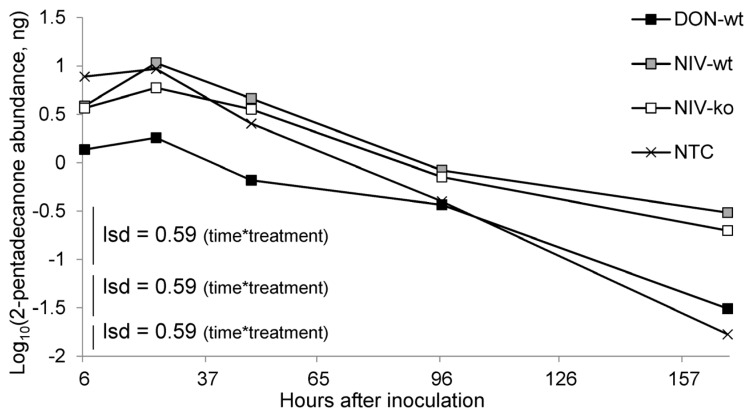
Change in abundance of 2-pentadecanone over time. Treatment was significant (*p* = 0.019) as was the interaction between treatment and time (*p* < 0.001). DON-wt: DON-producer; NIV-wt: NIV-producer; NIV-ko: *tri5* knock out transformant of NIV-wt; NTC: non-treated control. Greenhouse-Geisser ε = 0.68.

There were significant differences between treatments in the evolution of 2-pentadecanone over time in the host volatiles sampled (*p* = 0.006) (Figure 6). Over time, the abundance of 2-pentadecanone in all treatments peaked at 24 hours after infection (HAI) then decreased, but this drop in abundance was greatest in mock inoculated controls and the DON-wt treatment. Two of the three inoculated treatments, NIV-wt (0.305 ng·μL^−1^) and NIV-ko (0.198 ng·μL^−1^), produced greater amounts of 2-pentadecanone after a week of infecting the host compared to mock-inoculated controls (0.017 ng·μL^−1^). The abundance of 2-pentadecanone at 7 DAI in the DON-wt treatment (0.031 ng·μL^−1^) was not different from controls. The change in abundance over time of all other chemicals identified by peak enhancement GC/MS were not significantly affected by treatment, but time was a significant factor in all cases (*p* < 0.001). 

## 3. Discussion

Each of the *Fusarium* isolates in the study was characterised through analysis of DNA and mycotoxins. NIV-wt and NIV-ko mutants contained genetic sequences typical of NIV-producing isolates, and NIV-wt samples contained high levels of NIV but NIV-ko samples did not, thus confirming that this mutant had the expected difference in mycotoxin production. Thus, any differences between the NIV-wt and NIV-ko treatments could be attributed specifically to the presence/absence of NIV during infection. The DON-wt isolate was shown to possess genetic sequences of 15ADON-producers, and this trait was confirmed as the isolate produced DON during host infection. While comparing any results from the DON-wt and NIV-wt isolates, the different mycotoxins are likely to have a role in any observed effects, but it is worth noting that these isolates are not isogenic and, so, other factors associated with isolate pathogenicity and virulence cannot be discounted. 

Confirmation of isolate character was corroborated with visual disease assessment of infected plants. The highest disease severity was observed in toxin-producing treatments, whilst bleaching was greatest in the DON-producing treatment. Both DON and NIV were factors that promoted development of visible disease in host plants. 

The most prominent finding of this work is the novel discovery that aphids were attracted to volatiles from hosts inoculated with the NIV-producer. This is in contrast to host volatiles from the mycotoxin deficient mutant of the same isolate to which aphids were neither attracted nor repelled. Therefore, attraction can be linked to NIV production by the isolate. Furthermore, the attractiveness of the NIV-producer’s volatiles is also in contrast to the repellent nature of volatiles emitted by hosts infected with DON-producing isolates. This aphid behaviour is consistent with previous work where volatiles from hosts infected with a composite inoculum of three DON-producing *F. graminearum* isolates were also shown to be repellent to aphids [24]. The different effects of each mycotoxin on the host semiochemical output are detected by the aphids, and differential behavioural adaptations towards them have evolved. 

It has previously been shown that aphid mortality increases on hosts infected with DON-producing *F. graminearum* [24] and, as such, that the behavioural response to DON-producer infected host volatiles is one of avoidance; this behaviour is advantageous for aphid survival by evading an inhospitable environment. Whether aphid mortality would be affected on hosts infected with NIV-producing isolates is not known, and, so, we cannot conclude whether the aphids are being manipulated by the pathogen to be recruited to a poor host environment or whether the host environment is in fact not disadvantageous to the aphids and, therefore, avoidance would not be beneficial. Some *Fusarium* species are considered to be entomopathogenic [27], and the reactions of meal beetle larvae towards volatiles from wheat grain infected with different species followed a trend of avoidance of volatiles from entomopathogenic species and attraction or neutral response to non-entomopathogenic species, with the exception of *F. culmorum* [28]. Despite negatively impacting on meal beetle larvae populations, the insects were attracted to the volatiles from *F. culmorum* infected grain, highlighting that despite their attraction, the aphids tested here may still be negatively impacted on hosts infected with NIV-wt *F. graminearum*. The mechanism behind the generation of different host volatiles when infected by DON- and NIV-producing isolates of *F. graminearum* is also not known, although differences in phytotoxicity have been measured for DON and NIV, with DON being considered more phytotoxic [11] and leading to enhanced bleaching of spikes. Determining the mechanisms by which phytotoxicity differs would provide insight into the potential causes for the changes to host semiochemical production. 

The aphid-repellent host volatiles from DON-producing *F. graminearum* infection were determined previously to be a combination of six chemicals including 2-pentadecanone, 2-heptanone, 2-tridecanone, (-)α-cedrene, α-gurjunene, and phenyl acetic acid [24]. However, the chemical abundances of 2-heptanone and (-)α-cedrene did not differ significantly between treatments or over time for single treatments in the present study. Neither did these chemicals, or any others measured, differ in abundance between host volatiles from NIV-wt and NIV-ko infections. While these chemicals appear to be important as part of a blend of chemicals that elicit avoidance in aphids, their abundances alone are not linked to aphid behaviour or the mycotoxins present in the infected host. The effect of chemical composition in hosts infected with and without accompanying NIV accumulation remains unknown. Aphids have been shown to be capable of detecting chemicals at concentrations lower than the limit of detection by GC/MS [29]. Further electroantennography work should be performed to identify components of the volatile samples that aphids are able to detect, and accompanying behavioural assays are required to determine the attractive chemistry. Chemical abundances may be too simple a measure of identifying the subtle differences in chemical blends that combine to produce volatile signatures that are interpreted as being attractive to aphids. In previous work [24], the abundances of five out of the six aphid-detectable chemicals identified from DON-producing *F. graminearum* infected hosts were less than 1 ng·μL^−1^. Given that chemicals of very low abundance contribute to changes in behavioural responses by aphids, it can be appreciated why identification of chemicals from abundance data alone failed to produce suitable candidates for further investigation. The previous work also showed that several chemicals combined produced a repellent response, but components of the blend did not elicit a repellent response in aphids, and one, α-gurjunene, was slightly attractive [24]. A change in the proportion of α-gurjunene could be measured to reveal if this attractive component is increased in NIV-wt infected host volatiles. 

While the present study found *F. graminearum* infection by the DON-producing isolate to not consistently produce elevated 2-pentadecanone levels, as was observed previously [24], the remaining two pathogen treatments did produce elevated levels of 2-pentadecanone at 7 DAI compared to mock-inoculated controls. We speculate therefore that elevated 2-pentadecanone is unlikely to be affected by mycotoxin output by pathogens, but is more likely to be elevated as a consequence of toxin-independent pathogenesis of the host. The development of symptoms in infected hosts was rapid during headspace sampling, and the continued entrainment of inoculated spikes maintained high atmospheric moisture levels post-anthesis, which favours pathogen development [30]. Therefore, the evolution of host volatiles may not be representative of the time scale within which chemical evolution would occur following infection in the field. It is possible that due to the accelerated infection process in this experiment, the DON-wt treatment, which also incurred the highest level of disease severity and bleaching at 7 DAI, had ceased to emit the volatiles expected during pathogenesis. In extension of the earlier discussion regarding the nature of aphid olfaction, blends of chemicals may be of a greater importance than the crude abundance of any one component. 2-pentadecanone levels were lower at 7 DAI in the DON-wt compared to the NIV-ko treatment, despite the DON-wt VOCs eliciting a repellent response to aphids and the NIV-ko VOCs having a neutral effect. Despite the lower representation of 2-pentadecanone, which is itself repellent to aphids, additional chemicals present in the DON-wt are likely be responsible for maintaining the repellent effect on aphid behaviour that are not present in the NIV-ko VOCs. 

A low level of contamination was observed in the NIV-wt and NIV-ko infected plant material, as observed by both DON and 15ADON-chemotype DNA present in these treatments. The levels of this toxin were very low, (300- and 4000- fold lower than the concentration of DON in the DON-wt treatment for each of the NIV-wt and NIV-ko treatments, respectively), and appear to have not significantly impacted the hosts enough to alter their volatile production in such a way that would alter aphid interpretation of these signals. Even so, it cannot be discounted that the composition of the volatile chemicals produced in these treatments may have been altered by the effects of the contaminating DON-producer.

In summary, this work provides evidence of differential behaviour of aphids towards wheat plants with FHB caused by pathogens with differing mycotoxin production. This shows that mycotoxins can impact upon insect-interactions with infected hosts, achieved through subtle changes to host volatile chemistry which remain undefined. If this effect still occurs in the context of a field environment and volatile background, the expected outcomes of aphid infestation around the time of FHB infection could have differing consequences in geographical regions with different prevailing chemotypes of *F. graminearum* populations.

## 4. Materials and Methods 

### 4.1. Plant Material 

Wheat seeds (*T. aestivum* cv. Gallant) treated with 10 g of prothioconazole and 50 g clothianidin per 100 kg seed (Redigo Deter, Bayer, Cambridge, UK) were planted into compost (Levington’s F2 + S) in module trays (l × w × d: 2 cm × 2 cm × 5 cm) and vernalised for 6 weeks at 4 °C. Seedlings were then potted out into individual 5 L pots into compost (John Innes Type 2) and grown in a glasshouse under a 12 h photoperiod at 15–18 °C day and 12–15 °C night temperatures. At ear emergence (GS59) [31] plants were moved to a controlled environment chamber with 16 h/8 h day/night photoperiod, at 23 °C day and 15 °C night temperatures, and acclimated for one week prior to the application of treatments. 

### 4.2. Fusarium graminearum Isolates 

Isolates of *F. graminearum* used in these experiments were donated by the Schaefer group at Universitat Hamburg. They consisted of one DON-producing strain (FG8.1/#290), one NIV-producing strain (FG06/#293), and its *Tri5* knockout transformant (#294). The transformed strain was disrupted in the *Tri5* gene by vector insertion of a truncated *Tri5* fragment as previously described [18,32], and the knock-out success was previously verified by reverse transcriptase (RT)-PCR and ability to produce NIV or DON. Treatment names were abbreviated to DON-wt, NIV-wt, and NIV-ko for each of the DON- and NIV-producing wild types and the corresponding *tri5* knock-out transformant of the NIV-producing isolate. NTC denotes the non-treated controls.

### 4.3. Production of Fungal Inoculum and Inoculation of Wheat Ears

Isolates were grown in PDA for 10 days then subcultured on synthetic nutrient deficient agar (SNA) for a further 10 days. SNA plates were washed with 1 mL sterile distilled water (SDW) and agitated using a sterile l-shaped spreader. Spore suspensions were removed by pipette and the concentration calculated using a Neubauer improved haemocytometer (Marienfeld-Superior, Lauda-Königshofen, Germany). The concentration of the suspensions was adjusted to 2.5 × 10^5^ spores·mL^−1^ with SDW and kept for two days at 4 °C until inoculation. 

Wheat ears at anthesis were point inoculated on 10 spikelets per ear, by pipetting 10 μL spore suspension between the lemma and palea of individual spikelets. Control plants were inoculated in the same way using SDW. 

### 4.4. Experiment Replication

Three repeats of the inoculation experiment were performed, and four ears per plant were inoculated in the first and third experiments and two ears per plant in the second as limited numbers of same age tillers were produced. Two whole plant replicates were produced in the first and second repeats of the experiment, and four replicates were prepared in the third. After inoculation, ears were bagged in perforated plastic bags for 6 h to allow the inoculum to infiltrate the host tissues prior to volatile chemical collection. 

### 4.5. Volatile Chemical Collection and Olfactometer Bioassays

In the first two repeats of the experiment, 6 h after inoculation (HAI), a volatile chemical collection apparatus was connected to wheat ears. All inoculated ears of each plant were contained within odour-free cooking bags which were tied around the stems. Air was pumped into the bags from the base at a rate of 600 mL·min^−1^ and air was removed from the top corner, drawn out over a Porapak (TM) polymer matrix filter at a rate of 400 mL·min^−1^. Collections were commenced at 6, 24, 48, 96 HAI and 7 days after inoculation (DAI) and were carried out for approximately 24 h, with the exception of the 6 HAI collection which was only performed for 18 h. Between collections Porapak (TM) filters were removed, and volatile emissions were eluted into glass vials in 500 μL dichloromethane (DCM), and samples stored at −20 °C until use in olfactometer bioassays. Filters were cleaned between samples with 6 mL DCM. 

### 4.6. Disease Assessment 

On the eighth day after inoculation, after the final volatile collection, visual disease severity was assessed. The number of spikelets that displayed either dark lesions or bleaching was recorded as being symptomatic, and the disease severity was calculated as the percentage of symptomatic spikelets out of the total spikelets per ear. A second disease assessment was performed 14 DAI. The proportion of spikelets that were bleached out of the total number of spikelets per ear was calculated to give the severity of the bleaching symptoms at both 7 and 14 DAI. 

### 4.7. DNA Extraction and QPCR 

Ears were harvested 21 DAI and freeze-dried without threshing. Samples were then ground to flour using a commercial coffee grinder (Krups). DNA was extracted from flour samples (0.5 g) using CTAB buffer (3.75 mL), following a method that has been described previously [24]. DNA was resuspended in TE buffer (200 μL) by warming tubes in a hot block (65 °C) for 2 h and vortexed. The concentration of DNA in each sample was determined by spectrophotometry at wavelengths 260, 280, and 328 nm (Cary50 UV Spectrophotometer, Santa Clara, CA, USA) and recorded in software (Simple Reads). DNA stocks were then diluted to 20 ng·μL^−1^ and the concentration of diluted stocks confirmed by spectrophotometry. Due to the large quantities of target DNA being quantified after the first round of quantitative PCR, stocks were further diluted to 4 ng·μL^−1^ for use in *F. graminearum* and *Tri5* specific assays. 

DNA stocks were used in quantitative real-time PCR (QPCR) assays using four different primer sets, shown in Table 1. Primers for *F. graminearum*, NIV-producing isolates, 15-ADON producing isolates, and 3-ADON producing isolates were used. All assays used a total volume of 13 μL comprising 2.5 μL template DNA, SYBR Green 2X MaterMix and 250 nM of each forward and reverse primers. The temperature cycling programs for each assay are listed in Table 1. 

A standard curve of DNA of known concentration was prepared for each assay, using DNA from pure fungal cultures of either known DON-producing (#216) or NIV-producing isolates (#281) from the University of Nottingham collection for each of the assays that used 15ADON- or 3ADON- and NIV-producer specific primers, which were based on the *Tri12* gene. Assays using primers for *F. gramineraum* which are not specific to chemotype were run twice, using each of the previous standards in each run. Quantifications were found to be similar with both standards, and results presented are a mean of the quantifications of both assays. Ten-fold dilutions of DNA standards were assayed, ranging from 1 to 10^−6^ ng·μL^−1^. This linear regression was used to estimate the starting quantity of target DNA in samples. The limit of quantification for the *F. graminearum* assay was 1 × 10^−5^ ng·μL^−1^, and for all others the limit of quantification was 1 × 10^−4^ ng·μL^−1^. 

### 4.8. Mycotoxin Extraction and LCMS/MS 

To extract NIV, flour samples (0.5 g) were added to 15 mL tubes along with 4 mL methanol (70%) and shaken for four intervals of 20 s (MPBio FastPrep). Tubes were centrifuged at 2000× *g* for 5 min then 1 mL supernatant was added to a glass vial and spiked with ^13^C-labelled NIV as an internal standard to produce a final concentration of 20 μg·kg^−1^. Following this, samples were dried under nitrogen gas and redissolved in methanol (10%) and stored at −20 °C until analysis. On the day of analysis, samples were warmed to room temperature, liquid content was transferred to a microcentrifuge tube and centrifuged for 5 min at 13,000× *g*. The supernatant was then transferred to a new glass vial and then analysed by LCMS/MS. 

To extract DON, flour samples (0.5 g) were added to 15 mL tubes with 4 mL methanol (70%), spiked with ^13^C-labelled DON at a concentration of 20 μg·kg^−1^, and shaken for four intervals of 20 s (MPBio FastPrep). Tubes were centrifuged at 2000× *g* for 5 min and 2 mL supernatant diluted into PBS (23 mL). The diluted samples were filtered through glass microfiber paper (VWS). The filtrate was then added to a 20 mL syringe attached to a deoxynivalenol, zearalenone and T2 (DZT) MS-PREP column (R-Biopharm, Darmstadt, Germany) and allowed to pass through by gravity over a half hour period. The DZT column was washed by passing 20 mL deionised water through the column using the syringe plunger to maintain a flow rate of 5 mL·min^−1^, then dried by passing air over the column five times. Using a 2 mL syringe and plunger, 1 mL methanol (100%) was passed over the DZT column at a rate of one drop per second. During the passage of methanol over the column, the plunger was withdrawn to reverse the flow and draw liquid back up from the column three times to ensure maximum elution of the toxins contained on the column into the methanol. Methanol was evaporated off from the samples under a stream of nitrogen gas until dry and redissolved in 500 μL methanol (10%) to prepare samples for analysis by LCMS/MS. 

The LCMS/MS analysis was performed on an Agilent 1100 series LC system (Agilent Technologies, Waldbronn, Germany) coupled to a triple quadrupole Micromass Quattro Ultima V4.0 SP4 (Waters) and equipped with Luna^®^ C18 Å100 LC (5 μm, 250 mm × 3 mm) column (Phenomenex, Torrance, CA, USA). The flow rate was set at 0.5 mL·min^−1^ and the injection volume was 30 μL. Mobile phase A was 90% water with 10% methanol, mobile phase B was 100% methanol. For DON analysis, a linear binary gradient was applied from 0% to 100% phase B within 15 min. The content of phase B was held for 7 min and then lowered to 0% within 20 s followed by equilibration of the column for 5 min. For NIV analysis, a linear binary gradient was applied from 0% to 50% phase B over 7.5 min, and from 50% to 100% over a further 30 s. The content of phase B was held for 1 min then lowered to 0% over 1 min and followed by equilibration of the column for 5 min. 

Quantitative determination of all compounds was performed by operating the mass spectrometer (Waters Corporation, Milford, MA, USA) in ESI positive and negative ionisation mode. Optimized instrument settings include capillary voltage 3.5 kV, source temperature 100 °C, desolvation temperature 450 °C, desolvation gas flow rate of 672 L·h^−1^ cone voltage, a cone gas flow rate of 72 L·h^−1^, and multiplier 650 V. Parent/daughter ions for DON, ^13^C-labelled DON, NIV, and ^13^C-labelled NIV were detected at 265/217, 279/230, 281/191, and 295/198.7 m/z, respectively, with a dwell time of 0.1 s. MassLynx 4.0 software (Micromass UK Ltd, Wythenshawe, UK, 2002) was used for data acquisition and processing. Quantification of the samples was carried out using a matrix of standards prepared in-house. The limit of detection was determined for DON and NIV was 20 μg·kg^−1^.

### 4.9. Analysis of VOCs by GC-MS 

Headspace samples (4 μL) were analysed by GC-MS using a capillary GC column (50 m × 0.32 mm i.d. HP-1) fitted with a cold on-column injector which was coupled directly to a mass spectrometer (VG Autospec, Fisons Instruments, Manchester, UK). Ionization was by electron impact at 70 eV, 250 °C. The oven temperature was maintained at 30 °C for 5 min and then programmed at 5 °C·min^−1^ to 250 °C·min^−1^. Tentative GC-MS identifications were confirmed by peak enhancement with authentic samples on both the polar and nonpolar GC columns [33]. The peak area of confirmed chemicals was obtained from chromatograms and amounts were quantified using a known amount (100 ng) of authentic standards. 

### 4.10. Statistics

Analyses of the full treatment structure were performed by general analysis of variance (ANOVA), with the exceptions of cases where treatments contained zero values that were excluded from analyses. Experiments were not found to be significantly different, so, all data were combined and analysed together. Direct comparisons between toxin-producing isolates and their trichothecene-deficient counterparts was analysed by ad hoc comparison of means with a critical threshold of 5% (*p* = 0.05). Abundance of VOCs over time was analysed by repeated measurements in ANOVA. Disease severity and bleaching severity data were angularly transformed, and DNA, DON and volatile chemical abundance data was log_10_ transformed to normalise the residuals. Data from olfactometer assays were analysed by *t*-test, with a critical threshold of *p* = 0.05 for significance. All analyses were performed in Genstat v. 15.2 (VCN International, Harpenden, UK). 

## Figures and Tables

**Table 1:** Primers and programs used in quantitative polymerase chain reaction assays confirmation of treatment characters.

Assay Name	Target	Primer Sequences (5′–3′)	Thermocycling Programme	Product Size (bp)	Reference
*F. graminearum*	ITS	Fg16NF: ACAGATGACAAGATT CAGGCACA Fg16NR: TTCTTTGACATCTGTTCAACCCA	95 °C for 90 s; 35 cycles of 94 °C for 30 s, 64 °C for 45 s, 72 °C for 45 s; 72 °C for 5 min	280	[25]
NIV-producers	*Tri12*	NIV/f: GCCCATATTCGCGACAATGT NIV/r: GGCGAACTGATGAGTAACAAAACC	50 °C for 2 min; 95 °C 10 min; 40 cycles of 95 °C for 15 s, 60 °C for 1 min	77	[26]
15ADON-producers	*Tri12*	15ADONfwd: GTTTCGATATTCATTGGAAAGCTAC 15ADONrev: CAAATAAGTATCGTCTGAAATTGGAAA	50 °C for 2 min; 95 °C 10 min; 40 cycles of 95 °C for 15 s, 60 °C for 1 min	60	[26]
3ADON-producers	*Tri12*	3ADONf: AACATGATCGGTGAGGTATCGA 3ADONr: CCATGGCGCTGGGAGTT	50 °C for 2 min; 95 °C 10 min; 40 cycles of 95 °C for 15 s, 60 °C for 1 min	57	[26]

## References

[1] Goswami R.S., Kistler H.C. (2004). Heading for disaster: *Fusarium graminearum* on cereal crops. Mol. Plant Pathol..

[2] Waalwijk C., Kastelein P., De Vries I., Kerenyi Z., van der Lee T., Hesselink T., Kohl J., Kema G. (2003). Major changes in *Fusarium* spp. in wheat in The Netherlands. Eur. J. Plant Pathol..

[3] Xu X.M., Parry D.W., Nicholson P., Thomsett M.A., Simpson D., Edwards S.G., Cooke B.M., Doohan F.M., Brennan J.M., Moretti A. (2005). Predominance and association of pathogenic fungi causing Fusarium ear blight in wheat in four European countries. Eur. J. Plant Pathol..

[4] Parry D.W., Jenkinson P., McLeod L. (1995). Fusarium ear blight (scab) in small-grain cereals—A review. Plant Pathol..

[5] Lee T., Han Y.K., Kim K.H., Yun S.H., Lee Y.W. (2002). *Tri13* and *Tri7* determine deoxynivalenol- and nivalenol-producing chemotypes of *Gibberella zeae*. Appl. Environ. Microbiol..

[6] (2006). European Union Commission Regulation (EC) No. 1881/2006 setting maximum levels of certain contaminants in foodstuffs. Off. J. Eur. Union.

[7] Carter J.P., Rezanoor H.N., Holden D., Desjardins A.E., Plattner R.D., Nicholson P. (2002). Variation in pathogenicity associated with the genetic diversity of *Fusarium graminearum*. Eur. J. Plant Pathol..

[8] Van der Lee T., Zhang H., van Diepeningen A., Waalwijk C. (2015). Biogeography of *Fusarium graminearum* species complex and chemotypes: A review. Food Addit. Contam. Part A Chem. Anal. Control Expo. Risk Assess..

[9] Ward T.J., Clear R.M., Rooney A.P., O’Donnell K., Gaba D., Patrick S., Starkey D.E., Gilbert J., Geiser D.M., Nowicki T.W. (2008). An adaptive evolutionary shift in Fusarium head blight pathogen populations is driving the rapid spread of more toxigenic *Fusarium graminearum* in North America. Fungal Genet. Biol..

[10] Foroud N.A., McCormick S.P., Macmillan T., Badea A., Kendra D.F., Ellis B.E., Eudes F. (2012). Greenhouse studies reveal increased aggressiveness of emergent Canadian *Fusarium graminearum* chemotypes in wheat. Plant Dis..

[11] Eudes F., Comeau A., Rioux S., Collin J. (2000). Phytotoxicity of eight mycotoxins associated with *Fusarium* in wheat head blight. Canad. J. Plant Pathol.-Revue Canad. Phytopathol..

[12] Marin S., Ramos A.J., Cano-Sancho G., Sanchis V. (2013). Mycotoxins: Occurrence, toxicology, and exposure assessment. Food Chem. Toxicol..

[13] D’Mello J.P.F., Placinta C.M., Macdonald A.M.C. (1999). *Fusarium* mycotoxins: A review of global implications for animal health, welfare and productivity. Anim. Feed Sci. Technol..

[14] Suzuki T., Iwahashi Y. (2014). Phytotoxicity Evaluation of Type B trichothecenes using a *Chlamydomonas reinhardtii* model system. Toxins.

[15] Hohn T.M., Plattner R.D. (1989). Expression of the trichodiene synthase gene of *Fusarium-sporotrichioides* in *Escherichia-coli* results in sesquiterpene production. Arch. Biochem. Biophys..

[16] Kimura M., Tokai T., O’Donnell K., Ward T.J., Fujimura M., Hamamoto H., Shibata T., Yamaguchi I. (2003). The trichothecene biosynthesis gene cluster of *Fusarium graminearum* F15 contains a limited number of essential pathway genes and expressed non-essential genes. FEBS Lett..

[17] Proctor R.H., Hohn T.M., McCormick S.P. (1995). Reduced virulence of *Gibberella zeae* caused by disruption of a trichothecene toxin biosynthetic gene. Mol. Plant-Microbe Interact..

[18] Maier F.J., Miedaner T., Hadeler B., Felk A., Salomon S., Lemmens M., Kassner H., Schaefer W. (2006). Involvement of trichothecenes in fusarioses of wheat, barley and maize evaluated by gene disruption of the trichodiene synthase (*Tri5*) gene in three field isolates of different chemotype and virulence. Mol. Plant Pathol..

[19] Jansen C., Von Wettstein D., Schafer W., Kogel K.H., Felk A., Maier F.J. (2005). Infection patterns in barley and wheat spikes inoculated with wild-type and trichodiene synthase gene disrupted *Fusarium graminearum*. Proc. Natl. Acad. Sci. USA.

[20] Kang Z., Buchenauer H. (1999). Immunocytochemical localization of fusarium toxins in infected wheat spikes by Fusarium culmorum. Physiol. Mol. Plant Pathol..

[21] Horevaj P., Brown-Guedira G., Milus E.A. (2012). Resistance in winter wheat lines to deoxynivalenol applied into florets at flowering stage and tolerance to phytotoxic effects on yield. Plant Pathol..

[22] Gosman N., Steed S.A., Chandler E., Thomsett M., Nicholson P. (2010). Evaluation of type I Fusarium head blight resistance of wheat using non-deoxynivalenol-producing fungi. Plant Pathol..

[23] Drakulic J., Bruce T.J.A., Ray R.V. (2016). Direct and host-mediated interactions between *Fusarium* pathogens and herbivorous arthropods in cereals. Plant Pathol..

[24] Drakulic J., Caulfield J., Woodcock C., Jones S.P.T., Linforth R., Bruce T.J.A., Ray R.V. (2015). Sharing a host plant (Wheat *Triticum aestivum*) increases the fitness of *Fusarium graminearum* and the severity of Fusarium head blight but reduces the fitness of grain aphids (*Sitobion avenae*). Appl. Environ. Microbiol..

[25] Nicholson P., Simpson D.R., Weston G., Rezanoor H.N., Lees A.K., Parry D.W., Joyce D. (1998). Detection and quantification of *Fusarium culmorum* and *Fusarium graminearum* in cereals using PCR assays. Physiol. Mol. Plant Pathol..

[26] Nielsen L.K., Jensen J.D., Rodriguez A., Jorgensen L.N., Justesen A.F. (2012). *TRI12* based quantitative real-time PCR assays reveal the distribution of trichothecene genotypes of *F. graminearum* and *F. culmorum* isolates in Danish small grain cereals. Int. J. Food Microbiol..

[27] Gagkaeva T.Y., Shamshev I.V., Gavrilova O.P., Selitskaya O.G. (2014). Biological relationships between *Fusarium* fungi and insects (review). Sel'skokhozyaistvennaya Biologiya.

[28] Guo Z.Q., Doll K., Dastjerdi R., Karlovsky P., Dehne H.W., Altincicek B. (2014). Effect of fungal colonization of wheat grains with *Fusarium* spp. on food choice, weight gain and mortality of meal beetle larvae (*Tenebrio molitor*). PLoS ONE.

[29] Bruce T.J., Matthes M.C., Chamberlain K., Woodcock C., Mohib A., Webster B., Smart L.E., Birkett M.A., Pickett J.A. (2008). cis-Jasmone induces Arabidopsis genes that affect the chemical ecology of multitrophic interactions with aphids and their parasitoids. Proc. Natl. Acad. Sci. USA.

[30] Cowger C., Patton-Ozkurt J., Brown-Guedira G., Perugini L. (2009). Post-anthesis moisture increased Fusarium head blight and deoxynivalenol levels in North Carolina winter wheat. Phytopathology.

[31] Zadoks J.C., Chang T.T., Konzak C.F. (1974). Decimal code for growth stages of cereals. Weed Res..

[32] Maier F.J., Maiz S., Losch A.P., Lacour T., Schafer W. (2005). Development of a highly efficient gene targeting system for *Fusarium graminearum* using the disruption of a polyketide synthase gene as a visible marker. FEMS Yeast Res..

[33] Pickett J.A. (1990). Gas chromatography-mass spectrometry in insect pheromone identification three extreme case histories. Chromatography and Isolation of Insect Hormones and Pheromones.

